# Supplementary figures and images for: Impact of Shape Irregularity in Medial Sphenoid Wing Meningiomas on Postoperative Cranial Nerve Functioning, Proliferation, and Progression-Free Survival
Source: Cancers (Basel). 2023 Jun 7;15(12):3096. doi: 10.3390/cancers15123096 (PMC10296424; doi:10.3390/cancers15123096)

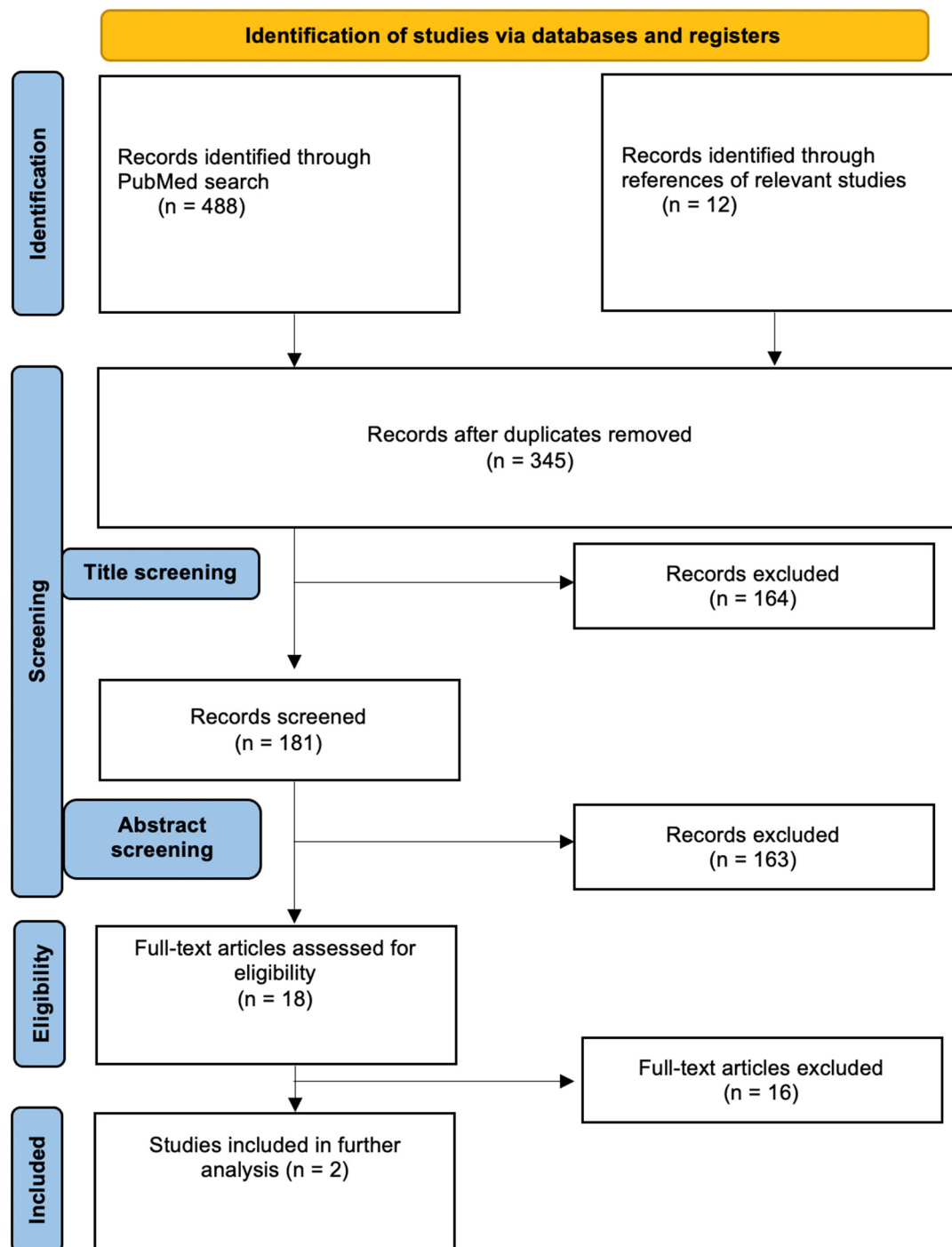

**Figure S1.** PRISMA Flowchart illustrating the literature search process.

Supplement: Supplementary file 1 [file cancers-15-03096-s001.zip › cancers-2398043-supplementary.pdf]
